# Factors that influence response classifications in chemotherapy treated patient-derived xenografts (PDX)

**DOI:** 10.7717/peerj.6586

**Published:** 2019-03-28

**Authors:** Joan E. Malcolm, Timothy M. Stearns, Susan D. Airhart, Joel H. Graber, Carol J. Bult

**Affiliations:** 1 The Jackson Laboratory, Bar Harbor, ME, United States of America; 2 Graduate School of Biomedical Sciences and Engineering, University of Maine, Orono, ME, United States of America; 3 The MDI Biological Laboratory, Bar Harbor, ME, United States of America

**Keywords:** Patient-derived xenograft (PDX), Treatment response, Dosing study design, Cisplatin, Docetaxel, PDX, Preclinical studies, Mouse models

## Abstract

In this study, we investigated the impact of initial tumor volume, rate of tumor growth, cohort size, study duration, and data analysis method on chemotherapy treatment response classifications in patient-derived xenografts (PDXs). The analyses were conducted on cisplatin treatment response data for 70 PDX models representing ten cancer types with up to 28-day study duration and cohort sizes of 3–10 tumor-bearing mice. The results demonstrated that a 21-day dosing study using a cohort size of eight was necessary to reliably detect responsive models (i.e., tumor volume ratio of treated animals to control between 0.1 and 0.42)—independent of analysis method. A cohort of three tumor-bearing animals led to a reliable classification of models that were both highly responsive and highly nonresponsive to cisplatin (i.e., tumor volume ratio of treated animals to control animals less than 0.10). In our set of PDXs, we found that tumor growth rate in the control group impacted treatment response classification more than initial tumor volume. We repeated the study design factors using docetaxel treated PDXs with consistent results. Our results highlight the importance of defining endpoints for PDX dosing studies when deciding the size of cohorts to use in dosing studies and illustrate that response classifications for a study do not differ significantly across the commonly used analysis methods that are based on tumor volume changes in treatment versus control groups.

## Introduction

The number of active compounds in oncology has quadrupled since 1996 and nearly doubled between 2008 and 2016 (Albrecht et al., 2018) to over 800 cancer drugs and vaccines in clinical trials or under review by the FDA. While growth in the number of compounds is key to improving therapy options for patients, retrospective studies have demonstrated a meager 5% to 20% success rate of cancer treatments entering clinical trials (DiMasi et al., 2013; Kola & Landis, 2004; Sharpless & Depinho, 2006); the remaining 80% to 95% of those compounds, which involved investment of significant resources to advance to a clinical trial, do not achieve approval or provide benefit to patients. Over 30% of the attrition has been attributed to a lack of efficacy uncovered in late-stage clinical trials (DiMasi et al., 2013). Preclinical models that reproducibly and accurately reflect drug efficacy and predict patient responses are also needed to eliminate ineffective compounds earlier in the development process (Johnson et al., 2001).

In addition to improving preclinical models, the methods of reporting of results also can promote reproducible, accurate, and comprehensive interpretation of results. Following guidelines such as ARRIVE (Animal Research: Reporting of *In Vivo* Experiments), which were developed to improve the design, analysis and reporting of research using animals, is critical to maximize information published and minimize unnecessary studies (Kilkenny et al., 2010).

Commonly used models in preclinical cancer therapy trials include *in vitro* cell lines, cell-line derived xenografts (CDXs), genetically engineered mouse models (GEMMs) and patient-derived xenografts (PDX). Cultured *in vitro* cell lines, and CDXs that are derived from them, have historically been the most broadly used *in vitro* cancer model. The limitations of these homogeneous cell line based models, and their alternatives, have been detailed in several reviews (Gillet, Varma & Gottesman, 2013; Wilding & Bodmer, 2014). Genetically-engineered mouse models (GEMMs) are generated through the introduction of targeted genetic alterations associated with specific human malignancies. The advantages offered by GEMMs include the presence of a functional immune system and intact tumor microenvironment, the ability to induce genetic aberrations at specific developmetal times in a tissue-specific manner, and the ability to study tumor intiation and progression (Heyer et al., 2010; Lee, 2014; Sharpless & Depinho, 2006). However, the precision required for the creation of GEMMs can be complex and technically challenging and may involve advanced genetic-engineering techniques that can result in “off-target” effects of gene editing with unintended consequences (Lee, 2014). Patient-derived xenograft models (PDXs) are generated by serial transplantation of human tumor cells or tissues in a mouse host (Abate-Shen et al., 2014). PDXs have been shown to more accurately reflect aspects of tumor biology observed *in situ* compared to cell culture and CDXs and are, therefore, a powerful tool for investigating complexities of cancer diseases with published correlations in histological phenotypes, genomic signatures, and treatment responses (Hidalgo et al., 2014; Kopetz, Lemos & Powis, 2012; Liu et al., 2010; Rosfjord et al., 2014; Tentler et al., 2012; Whittle et al., 2015). PDXs have been used previously to identify molecular targets of a compound, predict patient response and survival (Rosfjord et al., 2014; Tentler et al., 2012), and assess the response of a tumor to a compound at both the individual patient level (Garralda et al., 2014; Hidalgo et al., 2011; Izumchenko et al., 2017) and the population level (Gao et al., 2015). Limitations of PDX include the relative cost and long lead time of utilizing the model, relative to cell-line models. The time required to passage PDX model system to develop study animals with tumors at a treatable volume can take up to a year for some slower growing models. PDX are also heterogenous tumor fragments which provides a key advantage to the model, but how the tumors are divided and engrafted can also incorporate sampling bias across animal cohorts on study.

For PDXs to deliver on their promise as an effective platform for preclinical studies for cancer therapeutics, it is important to understand how different study design factors influence the reproducibility of treatment response classifications in this model system. Different methods of classifying treatment responses in xenografts have been proposed, but many have been evaluated only in cell lines and CDXs which lack the heterogeneity and subsequent growth variability supported by PDXs (Hather et al., 2014; Laajala et al., 2012; Liang, 2007; Wu & Houghton, 2010). For example, Hather et al. (2014) compared a tumor growth rate based method (RTC) to a traditional tumor volume treatment to control method (TC) using 219 CDXs and reported that RTC required fewer animals to achieve the same effectiveness as TC. They also determined that a cohort size of four animals achieved over 95% statistical power. To date, there have been no published studies demonstrating the relevance of the Hather study to PDXs.

In the study reported here we evaluated the impact of initial tumor volume, rate of tumor growth, cohort size, study duration, and analysis method on treatment response classifications in PDX models from The Jackson Laboratory (JAX) PDX Resource. The dataset used for this study consisted of cisplatin treatment response data for 70 PDX models representing ten cancer types with up to 28-day study duration using cohorts of 3–10 tumor-bearing mice. The analysis was repeated using docetaxel treatment response data for 40 PDX models.

## Methods

### PDX models

The data for this study were obtained from the JAX PDX Resource, under protocol 12027 approved by The Jackson Laboratory Institutional Animal Care and Use Committee (IACUC) before study initiation. All animal studies followed the IACUC guidelines. The models were generated using subcutaneous tumor implantation in female NOD.Cg-Prkdc^scid^ Il2rg^tm1Wjl^/SzJ (NSG; JAX strain 005557) host mice. Detailed methods for model generation are described elsewhere (Shultz et al., 2014). Information and data for PDX models from the JAX PDX Resource are publicly available from the PDX Portal hosted by Mouse Tumor Biology Database (MTB; http://tumor.informatics.jax.org/mtbwi/pdxSearch.do) (Krupke et al., 2017).

### PDX dosing study design

To assess the impact of study design factors on treatment response classification to chemotherapy agents, we analyzed drug response data from 70 cisplatin-treated PDX models representing ten cancer types and 40 docetaxel-treated PDX models representing ten cancer types from the JAX PDX Resource (Table S1).

Cisplatin and docetaxel are commonly used chemotherapeutic drugs used for treatment of numerous human cancers including bladder, head and neck, lung, ovarian, and testicular. Cisplatin and docetaxel interfere with DNA replication and repair mechanisms leading to apoptosis in dividing cells, but they do so via different mechanisms. Cisplatin is an alkylating agent and docetaxel is a taxane that interferes with microtubules.

For dosing studies, human tumor fragments (3–5 mm^3^) were implanted subcutaneously using a trocar into the right hind flank of NSG mice. Animals were monitored until the engrafted tumor reached approximately 200 mm^3^. Mice were then randomly categorized into study groups and treated with either 2.0 mg/kg IV cisplatin once per week for up to three weeks, 10.0 mg/kg IV docetaxel once per week for up to three weeks, or a vehicle control. The compound and dosing schedule for vehicle controls varied across studies because of other treatment groups that were run simultaneously with cisplatin or docetaxel. Most models (66) used 5.0 ml/kg IV 5% dextrose in water; the remaining used one of the following alternatives: (1) 10.0 ml/kg PO 0.5% CMC daily (3 models), (2) 5.0 ml/kg PO 2.5% DMSO in PBS daily (1 model), (3) 5.0 ml/kg IV combination of 5% dextrose in water and 0.5% CMC (1 model).

Clinical observations, body weights, and digital caliper tumor volume measurements were performed at least twice weekly for up to 28 days after study enrollment. Volume was calculated by using the following formula: volume = (length) × (width)^2^/2, in which the length is the larger of the two perpendicular axes and width is the smaller of two perpendicular tumor axes. Following the IACUC protocol, animals were removed from the study if tumors became visibly necrotic or if the animals demonstrated greater than 20 percent weight loss.

### PDX dosing study data

All models involved one treatment group and one control group evaluated for a minimum of 14 days, with 67 models enrolled for a minimum of three weeks and 53 for four weeks. Initial individual tumor volume ranged from 50 to 367 mm^3^ across all studies. The number of mice per cohort ranged from *N* = 3 to *N* = 11; 55 of the models had a treatment group with a minimum of *N* = 8 (Table S1). While the response was analyzed at four time points, the animal and tumor volume monitoring occurred two to three times per week for the duration of the study. Not all measurements used to calculate treatment responses at the end of a dosing study were available precisely on days 0, 14, 21, and 28. If a target timepoint did not have corresponding data, then the value that fell within the following range was used: day -1 to 1 grouped as day 0 or baseline volumes, 11 to 17 as day 14, 18 to 24 as day 21, and 25 to 31 as day 28. If multiple points fell within that range, then the closest day to 14, 21, or 28 was used. The study day of the control group follow-up was matched to the study day of the corresponding treatment group follow-up. Outliers were assessed using JMP v13.2.1; minor outliers were defined as 1.5 times the mean volume interquartile range, and major outliers were defined as three times the interquartile range, for both the treated and control groups on a specific measurement day. Outliers were included in all subsequent analyses, as there was no indication that they were the result of a technical error.

### Calculating treatment response

Table 1 lists the data analysis methods for treatment response that were evaluated in this study. For the study reported here, treatment response was assessed at three time points (14, 21, and 28 days, as available). Each of these methods incorporates a calculation of the ratio of treatment to control measures. The most frequently used method is based on the ratio between the average volume of the treatment group (V_T_) and the average volume of the control group (V_C_) on a specific day during the study, referred to as the tumor-to-control method (TC). The relative tumor volume (RTV) (Marangoni et al., 2007; Nemati et al., 2010; Xu et al., 2013), measure accounts for variation in initial volume of a tumor. Either the mean or median of the RTV of the treatment group (RTV_T_) is divided by the mean/median of the control group (RTV_c_). Neither the TC nor the RTV accounts for the rate of growth or measurements of volume beyond the final day of the study and the first day of the study. Hather et al. addressed this potential shortcoming by developing a rate-based method (RTC) that accounts for all the individual volume measurements throughout a study (Hather et al., 2014).

**Table 1:** Methods of assessing tumor response.

**Method**	**Abbreviation**	**Equation**
Traditional T/C	(TC)	$ \frac{{V}_{T}}{{V}_{C}} $
Relative tumor volume T/C	(RTV)	$ \frac{{ \left( \frac{{V}_{x}}{{V}_{0}} \right) }_{T}}{{ \left( \frac{{V}_{x}}{{V}_{0}} \right) }_{c}} $
Rate-based T/C	(RTC)	$1{0}^{ \left( {\mu }_{T}-{\mu }_{c} \right) xt}$

**Figure 1:**
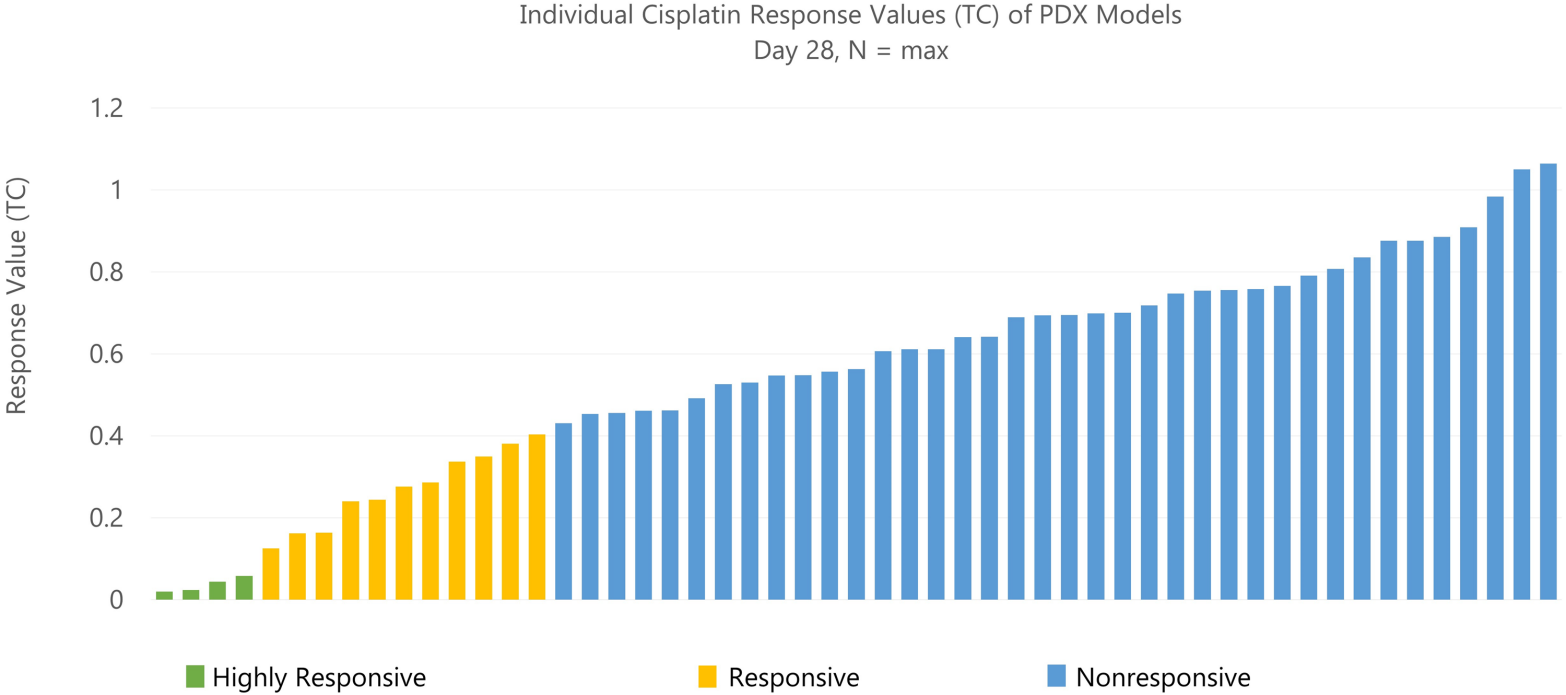
Distribution of response across models. A total of 53 PDX models treated with single-agent cisplatin had various response (TC) values at 28 days. Of 53 models with study length of 28 days, four PDX models were highly responsive (TC < 0.1) to cisplatin, 11 were somewhat responsive (0.1–0.42), and 38 were nonresponsive (>0.42).

Based on the tumor volume ratio of treated and controls calculated using the three methods described above, a response classification of “highly responsive” (ratio less than 0.1), “responsive” (ratio between 0.1 and 0.42), or “nonresponsive” (ratio greater than 0.42) was assigned. These criteria were adapted from the Drug Evaluation Branch of Cancer Treatment, NCI, which defines a T/C ≤ 0.42 as an active drug and T/C ≤ 0.10 as a highly active drug (Azmi et al., 2008; Geran et al., 1972; Huynh et al., 2008). The change in tumor volume was used as an indicator of effectiveness of the drug and as an indicator of drug activity. Figure 1 illustrates the distribution of T/C response at day 28 across 53 models treated with single-agent cisplatin. Of 53 models with a study length of 28 days, four PDX models were highly responsive to cisplatin, 11 were responsive, and 38 were nonresponsive.

To evaluate the impact of the number of replicates used to assign a response classification, we applied a bootstrapping (Mooney & Duval, 1993) technique, which randomly resamples the data with replacement to estimate parameters of a population empirically. By selecting subsets of both the treatment and control groups and using these to compute the response values of each of the three methods compared, we aimed to demonstrate how changes in study length and number of animals influenced the precision of the response values and classifications.

Utilizing only those models with the number of replicates per group (N) of greater than or equal to 8 for the duration of the study, 1,000 bootstrap replicates were performed for each subset from *N* = 3 to *N* = 7 relative to N = all, in which “all” refers to the maximum number of subjects available (8–10). The output determined the probability that the response of the subset N differed by: (1) > |0.05| of the N = all response value; (2) > |0.10| of the N = all response value; (3) change from N = all response classification. This method allows for replication of realistic sub-samples that may have been encountered if a smaller subset had been used in the study.

Statistical power analyses were run using the pwr package (version 1.2-2) in R (version 3.3.1) (R Core Team, 2016). Effect sizes were determined based on the TC/RTC/RTV (mean) /RTV (median) value using all available samples that that were nonresponsive (value of 0.42 or greater), responsive (value of 0.10 to 0.42) or highly responsive (value of less than 0.10). One thousand re-samplings were used to calculate new TC/RTC/RTV (mean) /RTV (median) values based on a subset of the original samples with replacement. The effect size was divided by the standard deviation of the resampled values. The sample size was determined as the number of subset samples used during the re-sampling process. An alpha value of 0.05 was established. Treatment response was calculated for TC/RTC/RTV (mean)/RTV (median) values, 0.42 and 0.10 for both directions (greater and less than) individually.

## Results

### Growth rate may impact treatment response classification more than initial tumor volume

**Figure 2:**
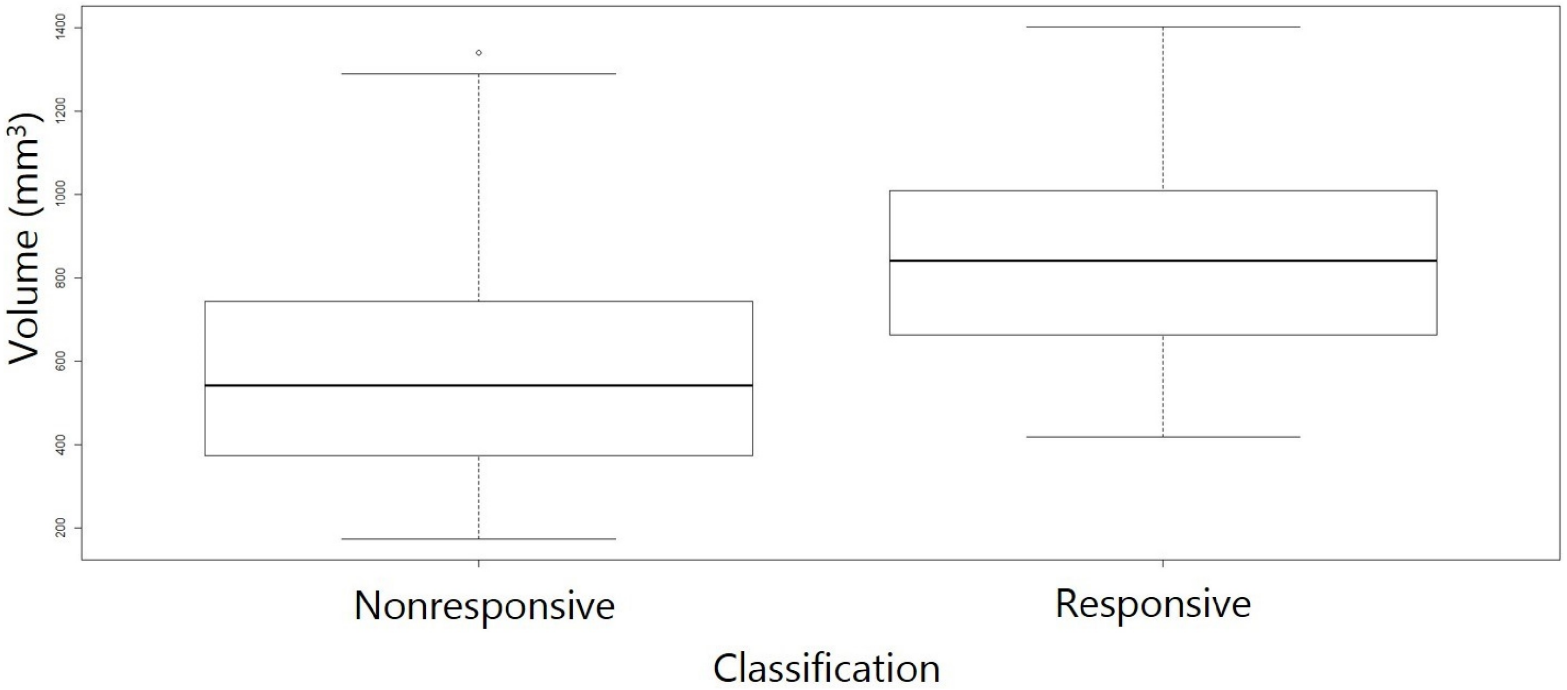
Day 21 control tumor volumes by response classified by RTC. Tumors that grew larger during the course of the study were more likely to result in a response classification. The average volume of the control tumors of the models (*R* = 0.36, *p* value = 0.01).

Table S2 lists the variables assessed in this study and their Spearman correlation coefficients with treatment response values. We observed that tumor volume of control samples at the end of the study had a moderate correlation value (*R* = 0.48) with response values across all methods which was most pronounced with the RTC data analysis. Figure 2 shows the control tumor volumes from study Day 21 of the cisplatin dosing studies. The average volume of the control tumors of the models that were classified as responsive were larger than those that were classified as nonresponsive indicating that the tumors that grew larger when unchallenged also were to be the ones that had a greater degree of response to the compound (*R* = 0.36, *p* = 0.01). The growth rate of the control tumors over the study duration was also moderately correlated (*R* = 0.35) to the response values. No other strong correlations (*R* > |0.30|) between the variables were identified.

### Calculating treatment response from mean tumor volume was more reproducible than from the median tumor volume

**Figure 3:**
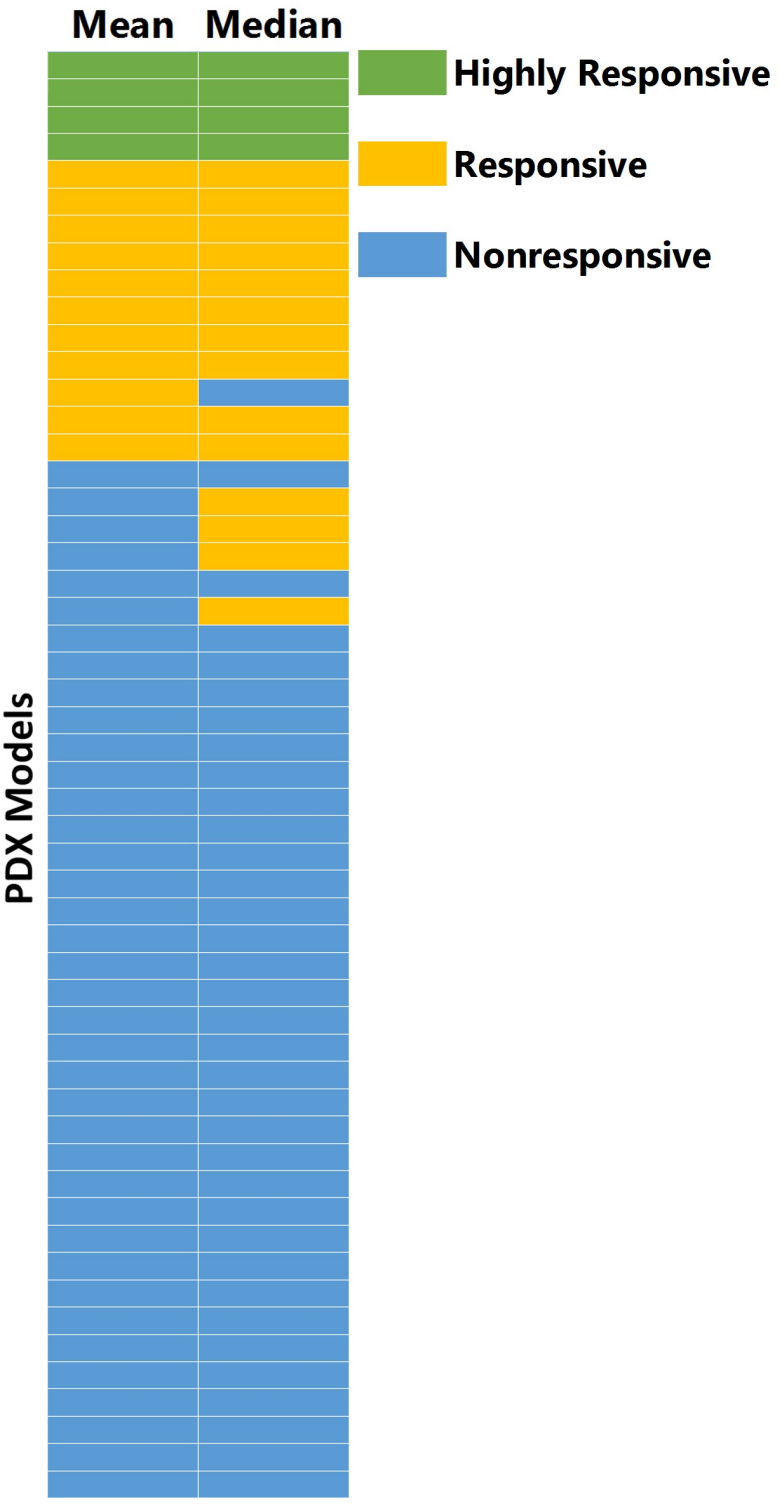
Comparison of treatment response values from median and mean tumor volumes. A total of 10% (five models of 50) of PDX models have a change in classification when calculating response with mean as opposed to median. The five models (TM00302, TM00214, TM00222, TM00185, TM01563) were all lung models, with four of the five models from primary tumors (not from tumors of metastatic or relapsed disease). Of the five models that differ in classification, mean is more sensitive for one model, TM00185, while median is more sensitive for four.

Figure 3 shows the comparison between response values that were calculated from the mean and median tumor volumes across the study groups. The power to detect the response (<0.42) was higher for mean compared to median for all three methods. Table 2 shows the power in detecting response versus the number of animals per group for RTV. Ten percent of the models assessed (five of 50) had a change in the response classification when calculating response with mean as opposed to median. Of the five models that differ in classifications, the mean was more sensitive for one model (TM00185), while the median was more sensitive for the other four. Two of these five models had major outliers (TM00185 in control group, TM00302 in cisplatin group); one model (TM00185) had four of eight control tumors not exceeding 500 mm^3^ over the duration of the study.

### Treatment response classification at 21-days is concordant with classification at 28-days

**Figure 4:**
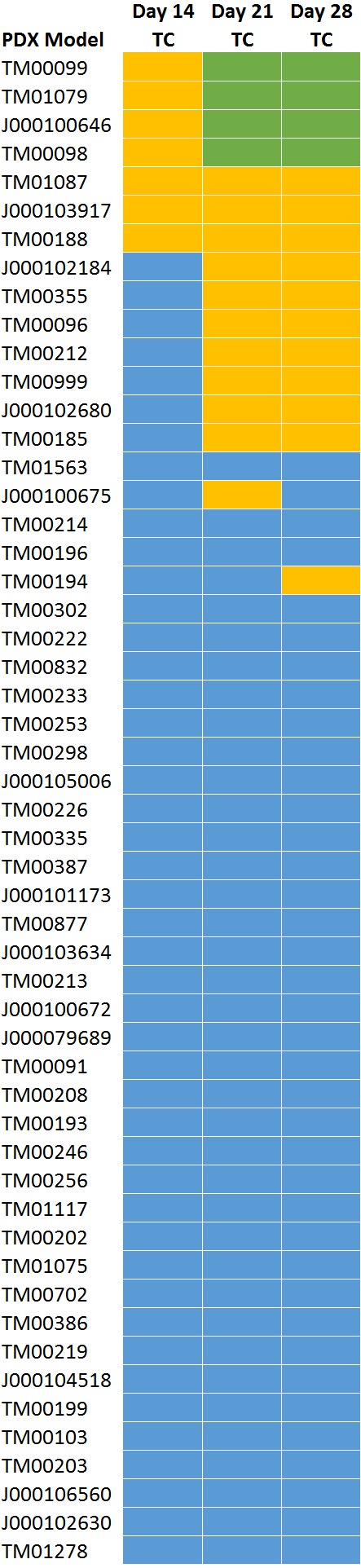
TC Response classification at study day 14, 21, and 28. All models classified as highly responsive or responsive on day 21 were consistent with those classified on day 28 with the exception of one model (TM00194).

Figure 4 shows the response classifications on study days 14, 21, and 28 for the three treatment response methods. All models classified as highly responsive or responsive on day 21 were consistent with those classified on day 28 with the exception of one model. While models that were classified as highly responsive on day 28 were observed to be responsive to cisplatin on day 14, a 14 day study duration would not have distinguished highly responsive and responsive models. None of the PDX models demonstrated a response to cisplatin by day seven.

**Table 2:** Mean Power of RTV classification when calculated with mean group volume and median group volume.

**Number of animals**	**Mean volume**	**Median volume**
3	0.770	0.716
4	0.826	0.796
5	0.869	0.811
6	0.881	0.852
7	0.963	0.903
8	1.000	0.986

### Treatment response classification does not vary by method of calculation

**Figure 5:**
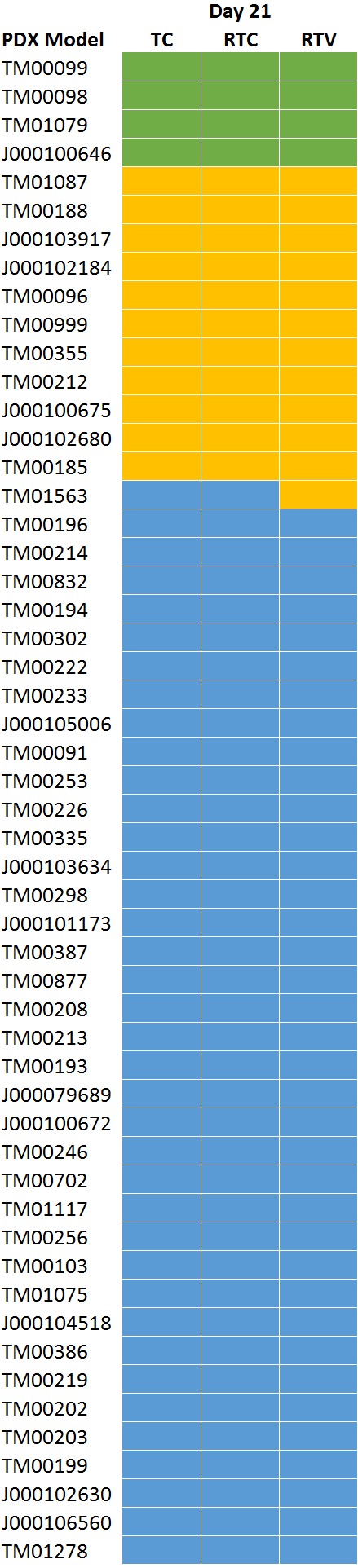
Response classification at Day 21 across three methods.

Table 2 displays a comparison of the treatment response values that were calculated for the three methods outlined in Table 1. Mean and median response values across all 53 models assessed were within 0.01 (1.7%) of each other for all methods. The standard deviation across models was minimal, but slightly higher in RTC. Figure 5 illustrates the classified response values determined by each of the methods using N = all at day 21. Only 1 (1.8%) of the models had a different response call among the methods.

### A cohort size of seven is required to achieve high statistical power

A probability analysis was performed to determine the frequency of changes in response classifications when a smaller N is used. Fifty-three models with PDX response data on day 21 with an N of 8 or greater were included in this analysis. Overall, the highly responsive and least responsive models have the lowest probability of a change in classification at lower N, suggesting that the ability to use a smaller cohort size depends on the degree of response that is of interest. The RTV method consistently classified models as highly responsive to cisplatin, with a 0% to 5% probability that the classification would differ from N of all when using an N of 3. The TC method consistently classified models as highly responsive to cisplatin (0% to 10% probability of the classification differing from N = all) for subset cohort sizes as low as *N* = 3. The highly responsive and least responsive models demonstrated the lowest probability of changing classifications at lower N.

**Figure 6:**
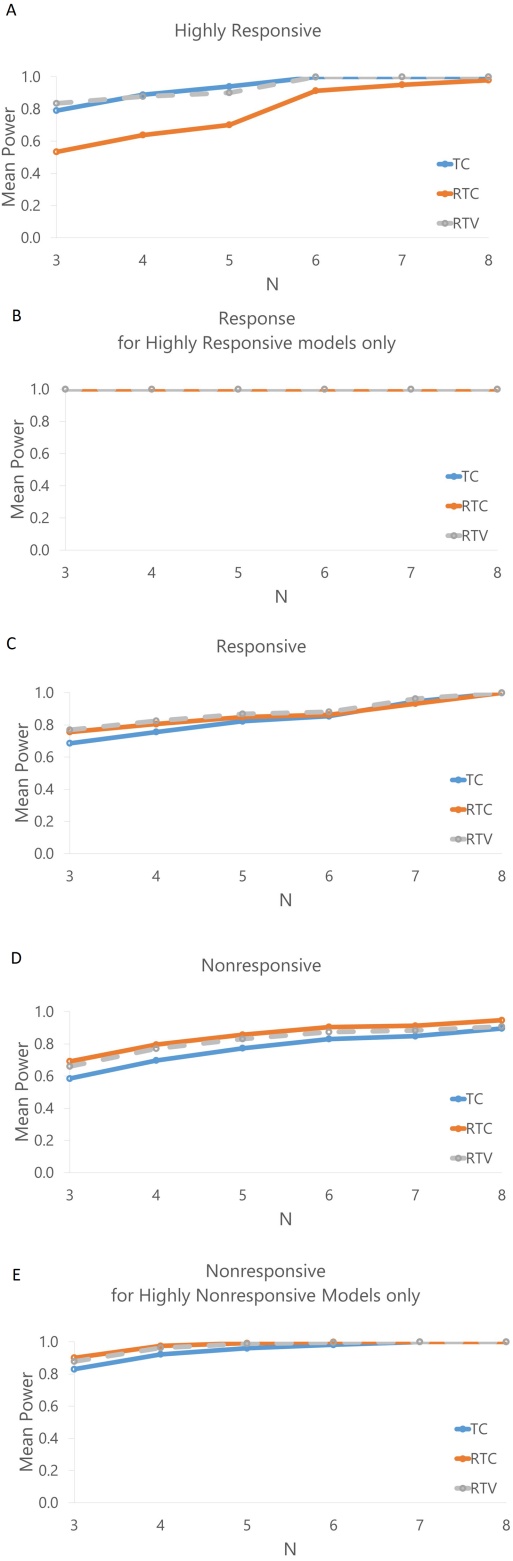
Mean power to detect cisplatin response classifications by number of animals used per group for each response method. (A) Mean power for highly responsive classifications. (B) Mean power for calling a response in models that are highly responsive (<0.10), showing that highly responsive models would be classified as responsive with 100% power. (C) Mean power for responsive models. (D) Nonresponsive models. (E) Mean power for non-responsive classifications of only those models that were highly nonresponsive (>0.80).

Figure 6 displays the mean effectiveness (as conveyed by the statistical power of the test) in classifying the model response. In general the effectiveness decreases as the number of animals per group decreases. Figure 6A demonstrates that the RTC method is the least effective for determining this classification, with effectiveness declining at an N of 3 (power of 0.534) relative to TC (0.791) and RTV (0.835). Both TC and RTV have an effectiveness of over 0.99 when an N of 8 is used; RTC’s effectiveness is also high, at 0.97. Figure 6B displays the effectiveness of identifying a response for the highly responsive models, which is 1.0 across all methods; this indicates that a highly responsive model would consistently be recognized as at least responsive with an N of 3. The statistical power in classifying nonresponsive models for TC, RTC, and RTV are 0.585, 0.692 and 0.661 respectively. The power in classifying highly nonresponsive (greater than 0.80) models as nonresponsive are 0.830, 0.901, and 0.878 respectively. Aside from RTC for nonresponsive classifications, the RTV method is consistently higher in effectiveness across categories and number of animals used. Correlations between power and growth rate, initial tumor volumes, and control tumor volumes were run. Only weak correlations (*R* < 0.30) were found; the largest correlations detected indicated that the standard deviation of the initial control tumor volume is negatively correlated with the mean effectiveness of nonresponse classifications (Spearman coefficient of −0.41).

While the highly responsive and nonresponsive models were assessed using an N of 3, a greater cohort size may be necessary to interpret response in models other than those with extreme response or nonresponse. The RTV was the most effective for classifying any responsive models with *N* = 3 at 77.0%, as illustrated in Fig. 6C, but an N of 7 was required to achieve above 90% statistical power (the chance of calling a treatment response when there is a treatment response to be detected), and an N of 8 was required to achieve an effectiveness rate of over 99.0%.

### Findings in studies of docetaxel produced similar results to those of cisplatin

Like the findings for cisplatin, the three methods demonstrated consistent docetaxel response classifications in 96% of models (22 of 23) and consistently classified the same models as highly responsive to docetaxel. Unlike cisplatin where no models demonstrated responses by day seven, three models had a docetaxel response classification computed for at least one of the three methods. Like cisplatin, the models highly responsive to docetaxel have minimal (less than 5%) probability of changing classifications with a cohort size of three animals. The RTV median method was the least effective in classifying docetaxel responses for 60% of models (12 of 20); in contrast, it was the most effective method for detecting cisplatin responses. The RTC method classified the lowest response values (was most sensitive) for docetaxel in 65% of models (13 of 20), suggesting that the sensitivity of the methods can be therapy-dependent.

## Discussion

In this study, we examined three commonly used methods for classifying treatment responses in PDX models and the impact of several study design factors on the consistency of those classifications. We showed that a cohort size of three is sufficient for identifying the four highly responsive and nine highly non responsive cisplatin treated tumors, suggesting that the use of a low cohort size to screen for chemotherapies that have a high degree of activity or models that have a high degrees of response is possible. However, it is important to note that cohort size depends on the study endpoints; distinguishing between the anticancer activity agents with similar tumor responses is not always possible with a low N. Achieving a balance between ensuring reproducible results and reducing cohort sizes and study duration will minimize the number of animals required and enable savings in laboratory resources.

We found that the average volume of the control groups of the models that were classified as responsive were larger than those that were classified as nonresponsive, indicating that the models that grew larger when unchallenged also had a greater degree of response to the compound. While this makes intuitive sense as a larger denominator in the treatment to control ratio would indicate a more robust treatment response, it may also suggest that a minimum tumor volume threshold is needed to assess response. This also highlights the importance of utilizing a control as results may be dependent upon the behavior of the tumor under normal/vehicle conditions as well as on the treatment.

Our results showed that a 21-day study duration is sufficient for reliable treatment response classification in studies focused on estimating drug efficacy. We observed that the same classifications that were discovered with a 28-day study duration could also be discovered for only a 21-day study duration for the drugs tested; this could enable faster distribution of data and greater savings of valuable lab resources. This finding is not consistent with the 14 day optimal duration shown by Hather, and we hypothesize that the lack of concordance is due to the differences in model system (CDXs vs PDXs). We note that as with cohort size, study duration is very dependent on endpoint criteria. For example, monitoring for acquired resistance or duration of response, will require longer study duration while dosing studies aimed at understanding infiltration of a compound into a tumor may require the tumors to be harvested and evaluated within hours or days after drug exposure.

Finally, we found that each of the methods consistently classifies responsiveness which suggests it is feasible to combine response data across studies that apply different methods assuming consistent response value thresholds have been used. The frequency of outliers in treatment response in PDX tumors has caused many researchers to consider the median to reduce the impact of outlying tumor measurements on the response classification (Carter et al., 2007; Hollingshead, 2008; Teicher, 2006), but our work shows that using the mean provides a more reproducible classification if no major outliers exist. Various enrollment criteria have also been described in the literature (Fiebig et al., 2007; Xu et al., 2013), but most frequently, an initial tumor volume group average of 200 mm^3^ is used(Garrido-Laguna et al., 2011; Jimeno et al., 2008; Krepler et al., 2017; Rubio-Viqueira et al., 2006; Yu et al., 2017; Zhang & Lewis, 2013), in addition to maintaining a small tumor volume distribution within a study group. We discovered that there is a non-statistically significant correlation between the standard deviation of the control tumor volumes at the beginning of the study and the effectiveness for nonresponsive classifications. Additional mice per cohort should be considered if tumor volumes vary within a study group.

## Conclusions

An important caveat to the work described in this manuscript is that our analyses were performed on less than a hundred PDX models and were limited to two chemotherapy agents; the findings may not extend to other drug classes. Other study design factors and analysis methods that could influence treatment responses in PDXs that were beyond the scope of this analysis and should be evaluated in future work. Orthotopically implanted PDXs, for example, were not considered but may be a factor in both engraftment success (Wang, Fu & Hoffman, 1992) and clinically relevant treatment responses to chemotherapy (Garrido-Laguna et al., 2011). Co-engraftment of fibroblasts and matrigel is also a method that varies across PDX studies, as does the use of hormone supplements such as estradiol for hormone-dependent cancers; the influence of these factors on study outcomes has not been investigated. The dosage and route of treatment agents were not considered here but could also be important factors in the consistency of treatment responses. With respect to methodology for calculating treatment response, we focused here on methods that compare treatment to control arms. We were specifically interested in determining if the findings described in Hather et al. (2014) performed on CDXs would apply to PDXs. We did not investigate response classification methods that are based on tumor regression or starting and ending values of the treated tumors only.

Ultimately, the value of PDXs as a preclinical platform will depend on how robustly treatment responses in these models translate to responses in patients. The results of our analyses do not speak directly to this important issue but they do support the robustness of treatment responses calculated using a typical PDX treatment study design and suggest that treatment responses can reasonably be compared across different studies that use an experimental design similar to the one described in this manuscript.

##  Supplemental Information

10.7717/peerj.6586/supp-1 Table S1 PDX treatment and control group characteristics in cisplatin and docetaxel studies PDX Model Characteristics.

10.7717/peerj.6586/supp-2 Table S1 Variables assessed and their Spearman correlation coefficients with treatment response values

10.7717/peerj.6586/supp-3 Table S3 Raw volume data of treatment and control subjects

10.7717/peerj.6586/supp-4 Supplemental Information 2 R code for bootstrapping technique selecting subsets of both the cisplatin treatment and control groups R code for bootstrapping technique selecting subsets of both the cisplatin treatment and control groups to compute the response values of each of the three classification methods. The output determined the probability that the response of the subset N differed by: (1) >|0.05| of the N = all response value; (2) >|0.10| of the N = all response value; (3) change from N = all response classification.

10.7717/peerj.6586/supp-5 Supplemental Information 5 R code for bootstrapping technique for docetaxel R code for bootstrapping technique selecting subsets of both the docetaxel treatment and control groups to compute the response values of each of the three classification methods. The output determined the probability that the response of the subset N differed by: (1) >|0.05| of the N = all response value; (2) >|0.10| of the N = all response value; (3) change from N = all response classification.

## Additional Information and Declarations

### Data Availability

The following information was supplied regarding data availability: http://tumor.informatics.jax.org/mtbwi/pdxSearch.do
Accession numbers: J000079689,J000080739, J000096652, J000099327, J000100646, J000100672, J000100674, J000100675, J000101173, J000102184, J000102630, J000102680, J000103634, J000103917, J000104256, J000104518, J000105006, J000106560, TM00090, TM00091, TM00096,TM00097, TM00098, TM00099, TM00103, TM00107, TM00185, TM00186, TM00188, TM00192, TM00193, TM00194, TM00196, TM00199, TM00202, TM00203, TM00208, TM00212, TM00213, TM00214, TM00219, TM00222, TM00226, TM00231, TM00233, TM00246, TM00253, TM00256, TM00298, TM00302, TM00335, TM00355, TM00356, TM00386, TM00387, TM00702, TM00784, TM00832, TM00877, TM00999, TM01029, TM01039, TM01075, TM01079, TM01087, TM01117, TM01149, TM01273, TM01278, TM01563.
